# Type 1 Kounis Syndrome Presenting as Coronary Pseudostenosis During Status Asthmaticus

**DOI:** 10.1016/j.jaccas.2026.110407

**Published:** 2026-09-30

**Authors:** Allen M. Yu, Billal Mohmand, Anita Z. Li, Qasim Jehangir, Rong Bai, Radha S. Gopalan, David G. Rizik, Paul Sorajja, Farouk Mookadam

**Affiliations:** a Division of Cardiovascular Diseases, Department of Internal Medicine, Banner University Medical Center Phoenix, University of Arizona College of Medicine-Phoenix, Phoenix, Arizona, USA; b Beijing Anzhen Hospital, Capital Medical University, Beijing, China; c Minneapolis Heart Institute Foundation at Abbott Northwestern Hospital, Minneapolis, Minnesota, USA

**Keywords:** acute coronary syndrome, acute heart failure, cardiac magnetic resonance, cardiomyopathy, echocardiography, myocardial infarction

## Abstract

**Background:**

Kounis syndrome is an allergic acute coronary syndrome that may produce multivessel coronary vasospasm, angiographically mimicking obstructive coronary artery disease without atherosclerosis.

**Case Summary:**

A 46-year-old atopic man admitted for status asthmaticus developed inferior ST-segment elevation myocardial infarction, complete heart block, ventricular fibrillation, and cardiogenic shock on hospital day 3. Emergent angiography revealed multivessel vasospasm with apparent stenoses up to 90%. Cardiac magnetic resonance showed ischemic injury without myocarditis. Repeat angiography on hospital day 20 demonstrated nonobstructive coronary artery disease, supporting vasospasm-mediated coronary pseudostenosis.

**Discussion:**

Severe status asthmaticus may precipitate type 1 Kounis syndrome. Apparent coronary stenoses during active vasospasm may represent vasospasm-mediated pseudostenosis and should be reassessed before revascularization is considered.

**Take-Home Messages:**

Severe status asthmaticus may precipitate type 1 Kounis syndrome with ST-segment elevation myocardial infarction, malignant arrhythmias, and cardiogenic shock despite nonobstructive coronary arteries. Apparent coronary stenoses during active vasospasm may represent vasospasm-mediated pseudostenosis and should be reassessed before revascularization is pursued.

## Visual Summary

**Figure:**
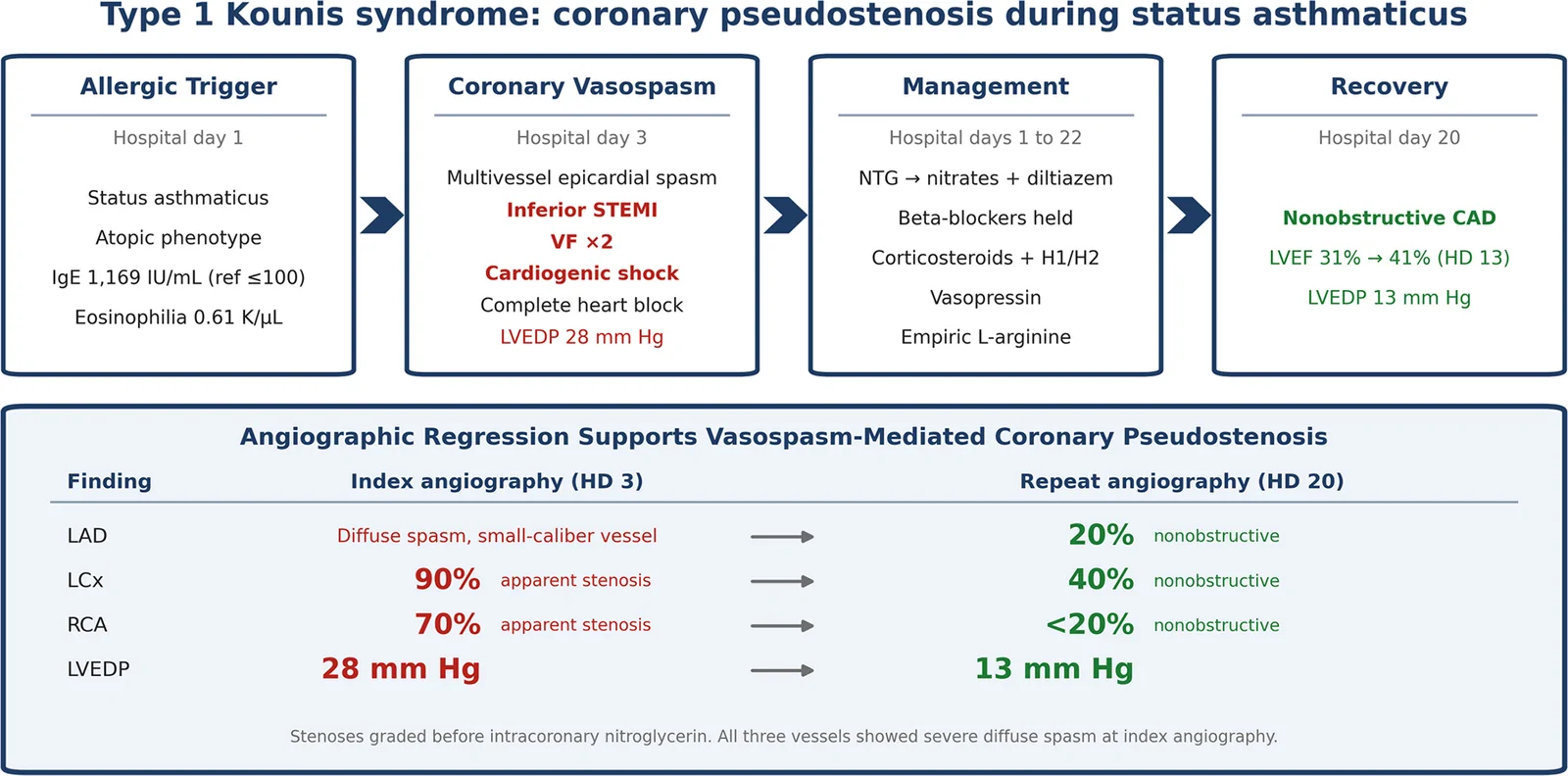

## History of Presentation

A 46-year-old man with asthma presented with progressive dyspnea and wheezing refractory to home albuterol therapy. Despite prehospital bronchodilator treatment, he arrived comatose (Glasgow Coma Scale score, 3) with severe hypercapnic respiratory acidosis on venous blood gas (pH 7.03, PCO_2_ 92 mm Hg), requiring emergent endotracheal intubation. He was admitted to the intensive care unit for status asthmaticus complicated by hypercapnic respiratory failure and vasopressor-dependent shock. On hospital day 3, telemetry demonstrated new bradycardia (51 beats/min) with inferior ST-segment elevation and complete heart block (Figure 1), prompting emergent coronary angiography.

**Figure 1:**
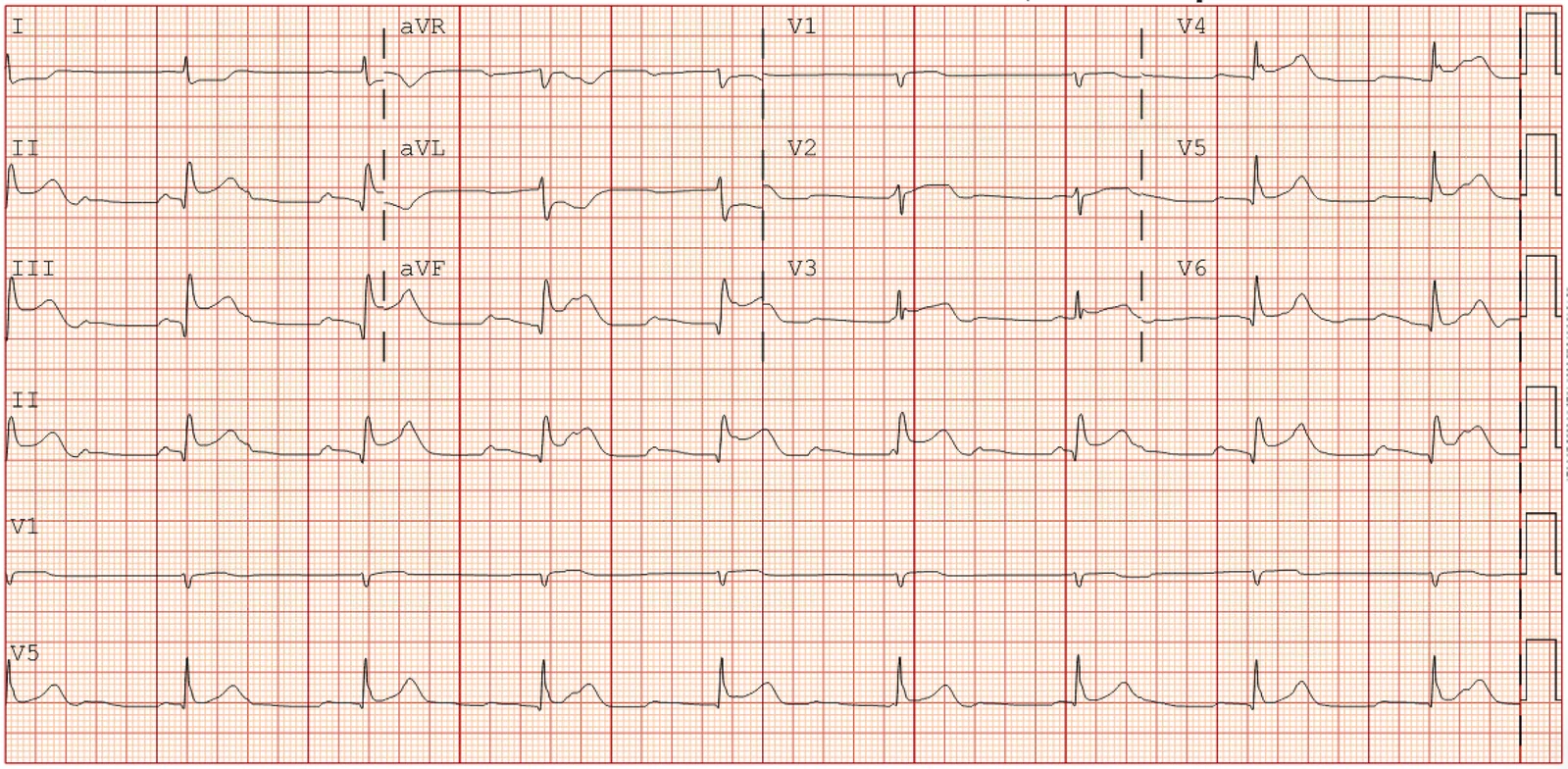
12-Lead Electrocardiogram Demonstrating Complete (Third-Degree) Atrioventricular Block With Atrioventricular Dissociation—P Waves Are Dissociated From the QRS Complexes (QRS Duration 105 ms)—and Acute Inferior ST-Segment Elevation in Leads II, III, and aVF With Minimal Anterior ST-Segment Elevation

## Past Medical History

The patient’s history included asthma with prior severe exacerbations, psoriasis, nicotine dependence, remote intravenous polysubstance use, and alcohol use disorder with recent relapse. He had no known cardiovascular disease or drug allergies.

## Differential Diagnosis

The differential diagnosis included obstructive acute coronary syndrome, diffuse coronary vasospasm (including Kounis syndrome),^1^^,^^2^ myocarditis, stress cardiomyopathy, and type 2 myocardial infarction secondary to hypoxemia or shock. Cocaine- and amphetamine-induced vasospasm were excluded after negative urine toxicology.

## Investigations

Transthoracic echocardiography demonstrated new biventricular systolic dysfunction with a left ventricular ejection fraction (LVEF) of 31% and reduced right ventricular systolic function. Emergent coronary angiography on hospital day 3 demonstrated a right-dominant coronary circulation with severe diffuse multivessel epicardial vasospasm involving the left anterior descending, left circumflex, and right coronary arteries. During active vasospasm, apparent 90% mid–left circumflex artery (LCx) and 70% mid–right coronary artery (RCA) stenoses created the angiographic appearance of severe multivessel obstructive coronary artery disease (Figure 2A, Video 1, Video 2, Video 3). Left ventricular end-diastolic pressure was 28 mm Hg. Intracoronary nitroglycerin produced immediate angiographic improvement, with the overall clinical and angiographic course summarized. High-sensitivity cardiac troponin T peaked at 179 ng/L on hospital day 3.

**Figure 2:**
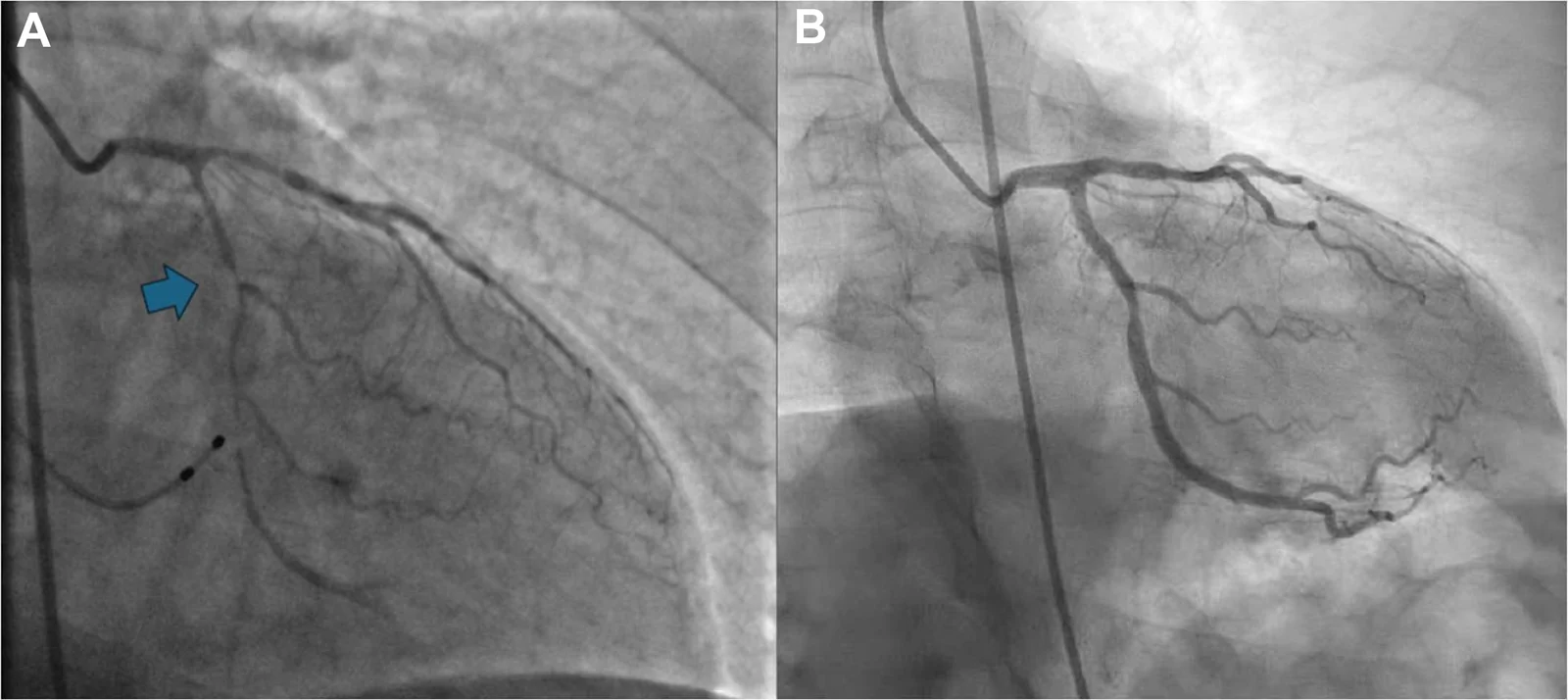
Angiographic Evidence of Vasospasm-Mediated Coronary Pseudostenosis (A) Index coronary angiography (hospital day 3) demonstrating severe diffuse multivessel epicardial vasospasm with apparent critical stenosis of the mid–left circumflex artery (approximately 90%, arrow) and diffuse vessel irregularity throughout the left coronary system; intra-aortic balloon pump markers are visible. (B) Repeat coronary angiography (hospital day 20) demonstrates marked improvement in vessel caliber with only moderate residual mid–left circumflex artery stenosis (approximately 40%), supporting transient vasospasm-mediated coronary pseudostenosis rather than fixed obstructive coronary disease.

The procedure was complicated by 2 episodes of ventricular fibrillation requiring defibrillation, complete heart block requiring transvenous pacing, and hemodynamic instability necessitating intra-aortic balloon pump support. Both devices were removed following hemodynamic stabilization (Figure 3).

**Figure 3:**
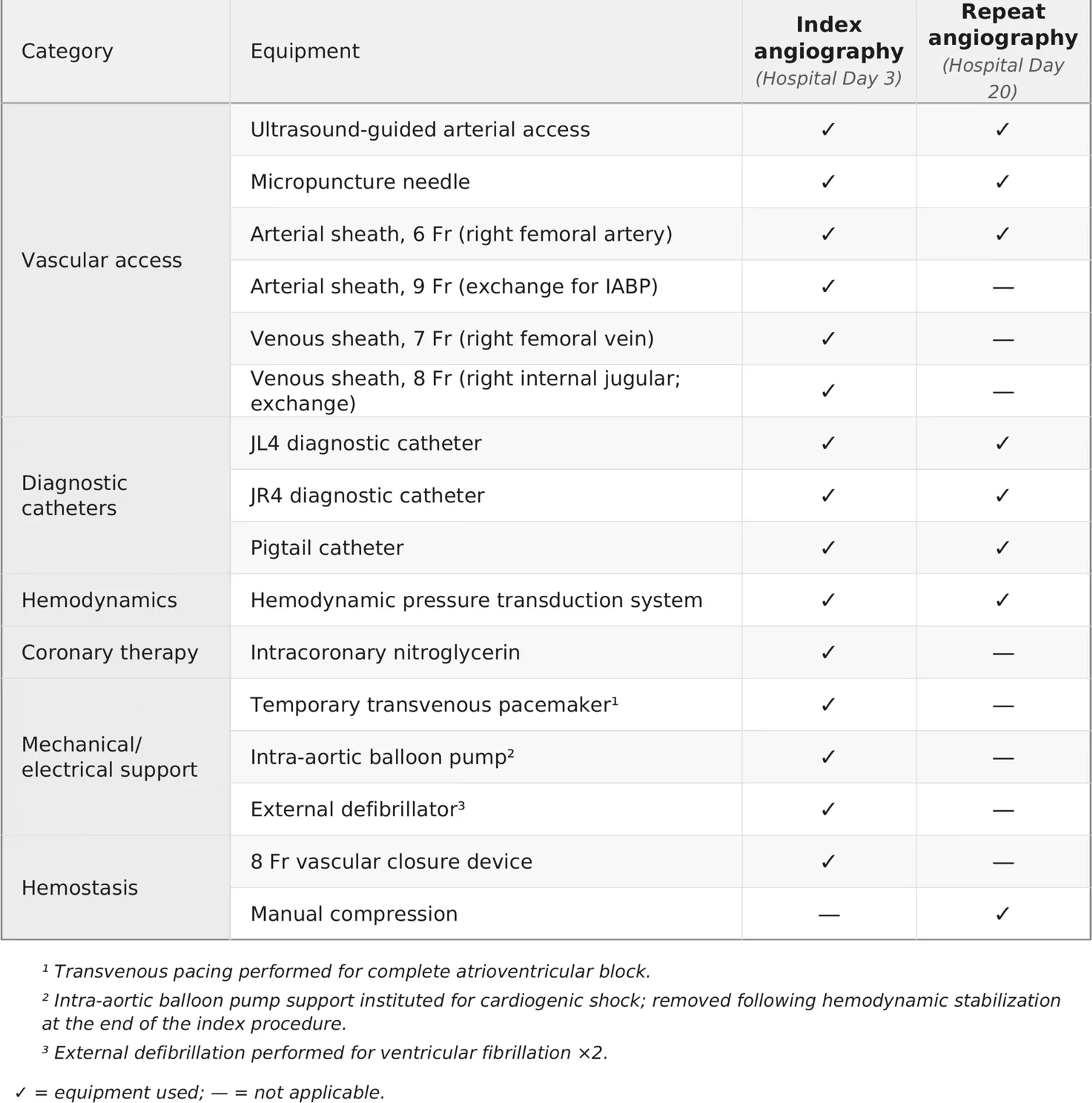
Equipment Used During Index and Repeat Coronary Angiography

**Visual Summary:**
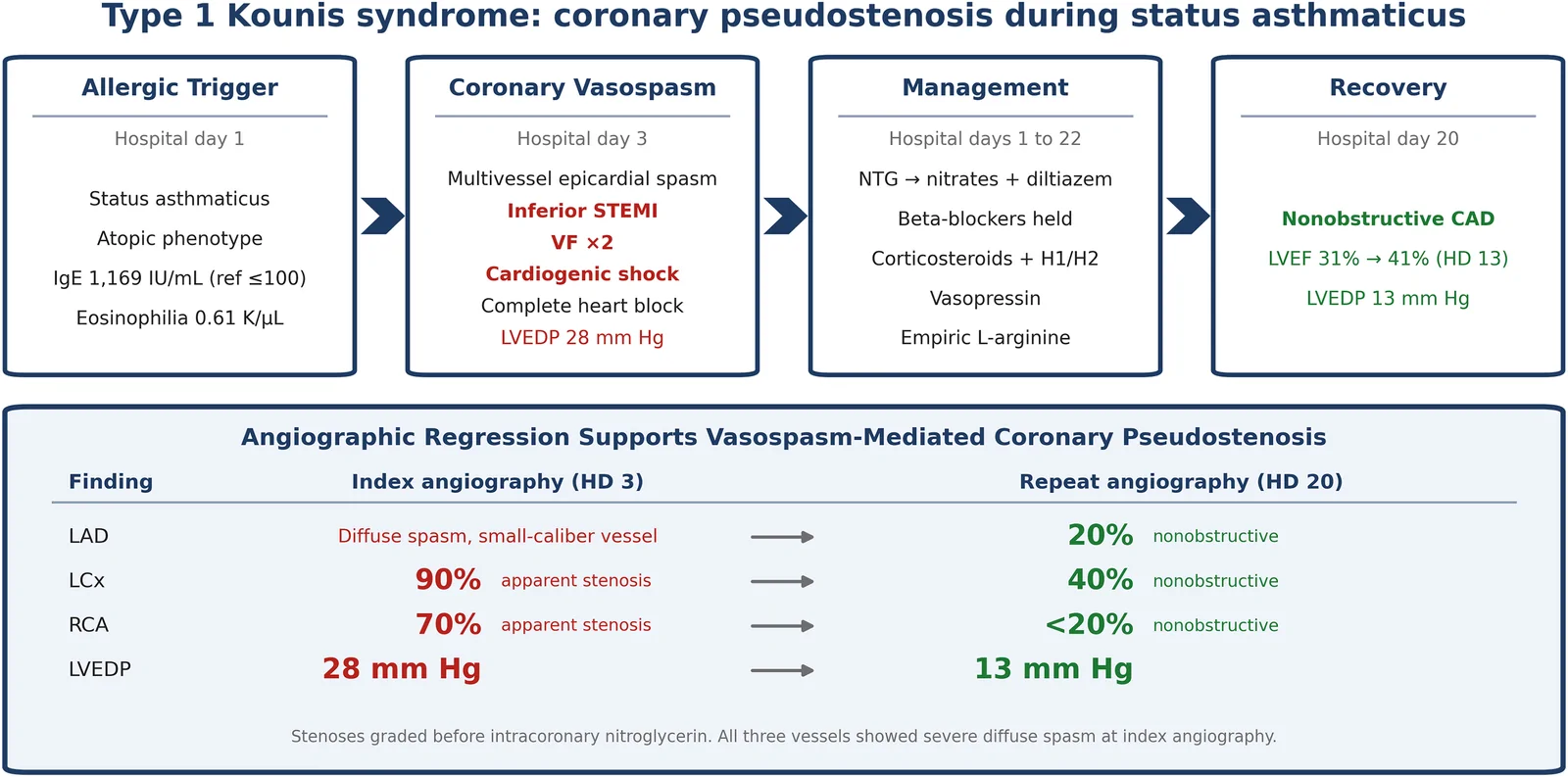
Pathophysiologic and Clinical Sequence From Severe Status Asthmaticus and Allergic Inflammation (Hospital Day 1) to Diffuse Multivessel Coronary Vasospasm, Malignant Arrhythmias, and Cardiogenic Shock (Hospital Day 3), With the Management Course (Hospital Days 1-22) and Angiographic Resolution (Hospital Day 20) The angiographic regression centerpiece demonstrates left ventricular end-diastolic pressure and apparent stenosis severity at index catheterization (hospital day 3) compared with repeat catheterization (hospital day 20), supporting transient vasospasm-mediated coronary pseudostenosis. L-arginine was used off-label for refractory vasospasm; postmyocardial infarction safety concerns are detailed in the Discussion. Left ventricular ejection fraction (LVEF) improvement from 31% to 41% reflects echocardiographic assessment on hospital day 13, preceding repeat angiography on hospital day 20. CAD = coronary artery disease; HD = hospital day; IgE = immunoglobulin E; LCx = left circumflex coronary artery; LVEDP = left ventricular end-diastolic pressure; NTG = nitroglycerin; RCA = right coronary artery; STEMI = ST-segment elevation myocardial infarction; VF = ventricular fibrillation.

Laboratory evaluation demonstrated leukocytosis with peripheral eosinophilia (0.61 K/μL before corticosteroid administration), supporting an atopic phenotype. Subsequent eosinopenia (0.00-0.01 K/μL) during hospitalization was consistent with glucocorticoid-induced suppression and acute illness physiology. Serum immunoglobulin E (IgE), first drawn on hospital day 4, was markedly elevated at 1,169 IU/mL (reference ≤100 IU/mL) and increased further to 1,963 IU/mL by hospital day 16, consistent with ongoing allergic activation. Serum tryptase obtained approximately 21 hours after the event was 3.1 μg/L, but delayed sampling limited its diagnostic utility.^3^

Cardiac magnetic resonance performed on hospital day 13 demonstrated extensive transmural late gadolinium enhancement involving the inferior, inferolateral, inferoseptal, and lateral walls, with a focal midlateral hypoenhanced region consistent with microvascular obstruction within the right coronary and left circumflex territories. The distribution—extensive transmural late gadolinium enhancement confined to the RCA and LCx territories—argued against inflammatory myocarditis and eosinophilic myocarditis, which typically produce a diffuse subendocardial pattern extending beyond coronary territories, and made Takotsubo cardiomyopathy unlikely.

Repeat transthoracic echocardiography on hospital day 13 demonstrated improvement in LVEF from 31% to 41%, with normalization of right ventricular function and right-sided filling pressures.

Repeat coronary angiography on hospital day 20 demonstrated only nonobstructive coronary artery disease with normalization of left ventricular end-diastolic pressure to 13 mm Hg. Apparent stenoses improved from 90% to 40% in the LCx and from 70% to <20% in the RCA, supporting transient vasospasm-mediated coronary pseudostenosis rather than fixed obstructive disease (Table 1, Figure 2B, Video 4, Video 5, Video 6). Provocative vasospasm testing was deferred given recent malignant ventricular arrhythmias.

**Table 1:** Angiographic and Hemodynamic Comparison Between Index and Repeat Catheterization

Finding	Index Cath (HD 3)	Repeat Cath (HD 20)
LAD	Severe diffuse spasm; small-caliber vessel	Approximately 20% mild nonobstructive disease
LCx	Severe diffuse spasm; apparent 90% stenosis	Approximately 40% moderate nonobstructive disease
RCA	Severe diffuse spasm; apparent 70% stenosis	<20% mild nonobstructive disease
LVEDP	28 mm Hg	13 mm Hg

The marked reduction in apparent stenosis severity between catheterizations supports transient vasospasm-mediated coronary pseudostenosis rather than fixed obstructive coronary disease. Angiographic stenoses reported before intracoronary nitroglycerin administration.

HD = hospital day; LAD = left anterior descending artery; LCx = left circumflex artery; LVEDP = left ventricular end-diastolic pressure; RCA = right coronary artery.

## Management

Norepinephrine and epinephrine were initiated on hospital day 1 for hemodynamic instability following intubation and were weaned off by hospital day 3, before the vasospastic event. Following the cardiac event, both were re-escalated, with vasopressin added to reduce catecholamine exposure despite its known coronary vasoconstrictive properties.^4^^,^^5^

Anti-ischemic therapy consisted of intravenous nitroglycerin followed by long-acting oral nitrates and diltiazem. Beta-blockers were withheld to avoid potentially exacerbating both coronary vasospasm and bronchospasm.^6^ Allergy-directed therapy included corticosteroids, H1/H2 blockade, and montelukast.

Refractory vasospasm despite nitrates and calcium-channel blockade prompted empiric L-arginine (3,000 mg 3 times daily) to target endothelial dysfunction, although evidence of efficacy in coronary vasospasm is limited, and postmyocardial infarction safety concerns are detailed in the Discussion.^7^, ^8^, ^9^ Guideline-directed medical therapy for heart failure with reduced ejection fraction was initiated, including sacubitril/valsartan, dapagliflozin, spironolactone, and high-intensity statin therapy.

## Outcome and Follow-Up

The patient recovered following control of coronary vasospasm and stabilization of respiratory failure. LVEF improved from 31% to 41% by hospital day 13. He was extubated on hospital day 11 and discharged home on hospital day 22 with long-acting nitrates, diltiazem, a prednisone taper, allergy-directed therapy, L-arginine, and guideline-directed medical therapy (sacubitril/valsartan, dapagliflozin, spironolactone, and high-intensity statin therapy).

Because ventricular fibrillation occurred during active coronary vasospasm, implantable cardioverter-defibrillator implantation was initially deferred, and a wearable cardioverter-defibrillator was prescribed at discharge. An implantable cardiac monitor was subsequently placed for arrhythmia surveillance, and no arrhythmias were documented at the time of reporting. Formal implantable cardioverter-defibrillator risk stratification and allergy and immunology evaluation remained pending.

## Discussion

Kounis syndrome is an underrecognized acute coronary syndrome triggered by allergic or hypersensitivity-mediated inflammatory activation.^1^^,^^2^^,^^4^ Although uncommon, it carries substantial morbidity, including ventricular arrhythmias and increased in-hospital mortality relative to allergic reactions without concomitant acute coronary syndrome.^10^

This case illustrates angiographically confirmed multivessel coronary pseudostenosis consistent with type 1 Kounis syndrome. Pretreatment peripheral eosinophilia and nitrate-responsive vasospasm supported the diagnosis. Serum IgE drawn on hospital day 4, after the cardiac event, was markedly elevated at a level consistent with pre-existing atopic sensitization. The 48-hour interval is atypical for classic Kounis syndrome, where events typically occur during or immediately after the allergic reaction. However, status asthmaticus itself involves sustained mast cell activation, while persistent allergic inflammation, evidenced by pretreatment eosinophilia and elevated IgE, may have maintained the coronary endothelium’s susceptibility to vasospasm. Cardiac magnetic resonance demonstrated transmural late gadolinium enhancement in coronary territories, arguing against inflammatory myocarditis. Transthoracic echocardiography demonstrated biventricular dysfunction with regional wall motion abnormalities, and the absence of documented apical or midventricular ballooning pattern argued against stress cardiomyopathy. Repeat angiography demonstrated marked regression of the apparent stenoses, supporting vasospasm-mediated coronary pseudostenosis in the absence of obstructive coronary artery disease. Alternative mechanisms, including catecholamine-mediated vasoconstriction, β-agonist therapy, and dynamic supply–demand mismatch, were considered.

Diffuse coronary vasospasm may simulate severe multivessel obstructive disease and prompt unnecessary revascularization. Coronary anatomy should be reassessed after vasospasm resolution when clinically feasible, as premature stenting risks treating a nonflow-limiting lesion and precipitating type 3 Kounis syndrome–related stent thrombosis.^6^ Management requires simultaneous targeting of coronary vasospasm and allergic inflammation. Nitrates and calcium channel blockers remained cornerstones of therapy, whereas β-blockers were avoided given concerns for exacerbating both coronary vasospasm and bronchospasm.^6^ Epinephrine was required for cardiogenic shock despite theoretical concern that α-1 adrenergic vasoconstriction may worsen coronary spasm, so vasopressin was added to reduce catecholamine exposure, though its benefit here remains unproven.^4^^,^^5^ A normal serum tryptase does not exclude the diagnosis, particularly when sampling is delayed, and diagnosis should integrate clinical context, atopic phenotype, temporal association, and angiographic findings.^3^^,^^4^ Limitations include delayed serum tryptase sampling beyond the recommended 90-minute to 4-hour window and the absence of a baseline level.^3^ Provocative vasospasm testing was deferred given recent malignant ventricular arrhythmias. As catecholamines were re-escalated for cardiogenic shock after catheterization on hospital day 3, α-1–mediated coronary vasoconstriction cannot be excluded. Off-label L-arginine was administered for refractory vasospasm given confirmed transmural myocardial infarction. The VINTAGE MI trial, which used the same dosing regimen, was terminated early because of excess mortality in the L-arginine arm (8.6% vs 0%; *P* = 0.01), and an American Heart Association scientific statement on complementary medicines advises against L-arginine following myocardial infarction.^8^^,^^9^ Any independent contribution of L-arginine to either recovery or adverse outcomes cannot be determined.

## Conclusions

Type 1 Kounis syndrome should be considered in atopic patients with ST-segment elevation myocardial infarction and diffuse multivessel coronary vasospasm. Apparent severe coronary stenoses during active vasospasm may reflect transient pseudostenosis rather than fixed disease. Repeat angiographic assessment after vasospasm resolution may prevent unnecessary revascularization, alongside integrated vasodilator and allergy-directed management.

**Take-Home Messages**

• Severe status asthmaticus in an atopic patient may precipitate type 1 Kounis syndrome with diffuse coronary vasospasm complicated by ST-segment elevation myocardial infarction, complete heart block, ventricular fibrillation, and cardiogenic shock despite nonobstructive coronary arteries.
• Apparent severe coronary stenoses during active vasospasm may represent transient vasospasm-mediated coronary pseudostenosis rather than fixed obstructive disease and should be reassessed after resolution of vasospasm before revascularization is pursued.

### Ethics Statement

This case report does not require institutional review board approval per *JACC:* *Case Reports* editorial policy for individual case reports.

## Funding Support and Author Disclosures

Dr Bai has received consulting fees from Synaptic Medical Corporation; travel, lodging, and food and beverage fees from Medtronic (MICRA) and Abbott Laboratories (AVEIR, ENSITE); and food and beverage fees from Boston Scientific Corporation (S-ICD System, FARAPULSE) and Biosense Webster (CARTO 3). Dr Gopalan has received research funding from Abbott Laboratories (HeartMate 3, CARDIOMEMS) and Nuwellis, Inc (Aquadex Smartflow Console); and food and beverage fees from Abbott Laboratories (HeartMate 3), ABIOMED, AstraZeneca (WAINUA), Merck Sharp & Dohme (WINREVAIR), BridgeBio Pharma (Attruby), Bayer Healthcare (Kerendia), Cardiac Dimensions, Haemonetics, ZOLL Services LLC (LifeVest), and Kestra Medical (Assure WCD). Dr Rizik has received speaker and faculty compensation from Medtronic, Inc (COREVALVE EVOLUT R) and Siemens Medical Solutions USA, Inc (ACUSON Origin Ultrasound System); consulting fees from Abbott Laboratories (MITRACLIP, PRESSUREWIRE); travel, lodging, and food and beverage fees from Medtronic, Inc (COREVALVE EVOLUT R), Siemens Medical Solutions USA, Inc (Artis icono floor, ACUSON SC2000), and Edwards Lifesciences Corporation (EVOQUE, SAPIEN 3); food and beverage fees from Abbott Laboratories (MITRACLIP, NAVITOR, PORTICO, PRESSUREWIRE, CARDIOMEMS), Teleflex LLC (GUIDELINER, MANTA, ORSIRO MISSION), Boston Scientific Corporation (WATCHMAN FLX), ACIST Medical Systems, Inc (CVI Systems), HeartFlow, Inc (FFRct), Medtronic, Inc (CARDIOBLATE), ZOLL Services LLC (LifeVest), Novartis Pharmaceuticals Corporation (LEQVIO), Shockwave Medical, Inc (Shockwave IVL System C2), and MIMEDX Group, Inc; and associated research funding from Boston Scientific Corporation (ACURATE IDE), Abbott Laboratories (SPIRIT 48, SUMMIT, TRILUMINATE Pivotal, AMPLATZER PFO Postapproval Study, XIENCE Skypoint Large Vessel, Tendyne), Procyrion, Inc (DRAIN-HF), Janssen Research and Development, LLC (LIBREXIA-ACS), Medtronic, Inc (EXPAND II), Edwards Lifesciences Corporation (JOURNEY), and Shockwave Medical, Inc (COSIRA-II). Dr Sorajja has consulting/advisory relationships with 4C Medical, Abbott Structural, Adona, AMX Technologies, Arcos, Boston Scientific, ConKay, Coramaze, CroiValve, Cultiv8, Edwards Lifesciences, Egg Medical, EvolutionMed, Foldax, GE Medical, Haemonetics, InQ8, LAZA, Medtronic, Mirus, Philips, Polares, Tricares, vDyne, Unorthodox Ventures, Valcare, and xDot. Dr Mookadam has received food and beverage fees from Novartis Pharmaceuticals Corporation (LEQVIO, ENTRESTO) and Pfizer Inc (VYNDAQEL). All other authors have reported that they have no relationships relevant to the contents of this paper to disclose.

## Appendix

Video 1 Index Coronary Angiography (Hospital Day 3)—Left Coronary Artery Severe diffuse epicardial vasospasm of the left anterior descending and left circumflex arteries, with apparent critical mid–left circumflex artery stenosis (approximately 90%) during active vasospasm before intracoronary nitroglycerin administration

Video 2 Index Coronary Angiography (Hospital Day 3)—Right Coronary Artery Severe diffuse vasospasm with apparent midvessel stenosis (approximately 70%) during active vasospasm before intracoronary nitroglycerin administration

Video 3 Index Coronary Angiography (Hospital Day 3)—Aortic Root Angiogram Diffuse multivessel coronary vasospasm with significant improvement following intracoronary nitroglycerin administration, supporting a vasospastic rather than fixed obstructive mechanism. Residual apparent stenoses in the mid–right coronary artery and mid–left circumflex artery are visible after nitroglycerin.

Video 4 Repeat Coronary Angiography (Hospital Day 20)—Left Coronary Artery, RAO Caudal Projection Marked improvement in vessel caliber compared with the index catheterization; residual moderate mid–left circumflex artery stenosis (approximately 40%) is present, representing a significant reduction from the apparent 90% stenosis observed during active vasospasm, supporting transient vasospasm-mediated coronary pseudostenosis.

Video 5 Repeat Coronary Angiography (Hospital Day 20)—Left Coronary Artery, LAO Caudal Projection Mild proximal and mid–left anterior descending artery stenosis (approximately 20%) without the diffuse vasospasm present at the index catheterization, confirming nonobstructive coronary artery disease

Video 6 Repeat Coronary Angiography (Hospital Day 20)—Right Coronary Artery, LAO Projection Marked improvement from the apparent 70% midvessel stenosis observed during active vasospasm to diffuse mild (<20%) nonobstructive disease, supporting transient vasospasm-mediated coronary pseudostenosis rather than fixed obstructive coronary disease
